# Non-Drug and Non-Invasive Therapeutic Options in Alzheimer’s Disease

**DOI:** 10.3390/biomedicines13010084

**Published:** 2025-01-01

**Authors:** Alina Simona Șovrea, Adina Bianca Boșca, Eleonora Dronca, Anne-Marie Constantin, Andreea Crintea, Rada Suflețel, Roxana Adelina Ștefan, Paul Andrei Ștefan, Mădălin Mihai Onofrei, Christoph Tschall, Carmen-Bianca Crivii

**Affiliations:** 1 Morpho-Functional Sciences Department, Iuliu Hațieganu University of Medicine and Pharmacy, 400347 Cluj-Napoca, Romania; simona.sovrea@umfcluj.ro (A.S.Ș.); annemarie.chindris@umfcluj.ro (A.-M.C.); sufletel.rada@umfcluj.ro (R.S.); lupean.roxana@umfcluj.ro (R.A.Ș.); madalin.onofrei@umfcluj.ro (M.M.O.); bianca.crivii@umfcluj.ro (C.-B.C.); 2 Molecular Sciences Department, Iuliu Hațieganu University of Medicine and Pharmacy, 400347 Cluj-Napoca, Romania; eleonora.dronca@umfcluj.ro (E.D.); andreea.crintea@umfcluj.ro (A.C.); 3 Radiology and Imaging Department, Emergency County Hospital Cluj, 400347 Cluj-Napoca, Romania; stefan_paul@ymail.com

**Keywords:** Alzheimer’s disease, non-drug, non-invasive, therapeutic options, FUS, TPS, ADSCs

## Abstract

Despite the massive efforts of modern medicine to stop the evolution of Alzheimer’s disease (AD), it affects an increasing number of people, changing individual lives and imposing itself as a burden on families and the health systems. Considering that the vast majority of conventional drug therapies did not lead to the expected results, this review will discuss the newly developing therapies as an alternative in the effort to stop or slow AD. Focused Ultrasound (FUS) and its derived Transcranial Pulse Stimulation (TPS) are non-invasive therapeutic approaches. Singly or as an applied technique to change the permeability of the blood–brain–barrier (BBB), FUS and TPS have demonstrated the benefits of use in treating AD in animal and human studies. Adipose-derived stem Cells (ADSCs), gene therapy, and many other alternative methods (diet, sleep pattern, physical exercise, nanoparticle delivery) are also new potential treatments since multimodal approaches represent the modern trend in this disorder research therapies.

## 1. Introduction

Alzheimer’s disease (AD) is a brain disorder, with age as a primary risk factor. In 2019, Alzheimer’s Disease International (ADI) estimated that over 50 million people worldwide were suffering from AD, and by 2050, the prevalence will increase by approximately three times. Every three seconds, an individual is diagnosed with dementia, and the annual costs for diagnosis and treatment are estimated at USD 1 trillion, a number that will probably double by 2030 [1]. The high prevalence of AD causes a huge financial burden on the health system and raises questions concerning the possible stigmatization of affected individuals.

AD develops gradually, with difficulties remembering recent events, associated with disorientation, language and thinking problems, mood swings, and a decline in personality and movement, which interfere with daily routines. After diagnosis, the life expectancy is five to ten years [2].

The disease has several important features, such as slow but progressive neurodegeneration, accumulation of extracellular β-amyloid (Aβ), intracellular neurofibrillary tangles (NFTs) of tau protein, loss of synapses, and increased inflammation [3].

In the majority of cases (95%), the disease is considered to be sporadic and late-onset. The origin of the disease in these cases is multifactorial, involving a complex genetic profile and environmental factors; some risk factors are well known, including aging, poor education, a personal history of cardiovascular diseases, depression, and the presence of the ɛ4 allele of the *apolipoprotein E* (*APOE*) gene. Yet, recent studies have revealed additional factors contributing to AD pathogenesis. Neuroinflammation has emerged as a critical mechanism, with evidence showing that chronic activation of microglia and astrocytes exacerbates neuronal damage and promotes Aβ plaque accumulation. Moreover, the loss of synaptic function and connectivity—driven by tau pathology and neuroinflammation—has been identified as a major determinant of cognitive decline in AD [4]. Advancements in imaging and fluid biomarkers have significantly enhanced the early diagnosis of AD. For example, positron emission tomography (PET) tracers specific to tau and amyloid are now allowing the visualization of these pathologies in vivo, while cerebrospinal fluid (CSF) and blood-based biomarkers, such as phosphorylated tau (p-tau) and neurofilament light chain (NfL), are proving effective for identifying preclinical stages of the disease [4].

The rest of the cases (5%) are hereditary due to mutations with high penetrance in three genes: *presenilin genes 1* and *2* (*PSEN1* and *PSEN2*), and, less commonly, the *APP* (*Aβ amyloid beta A4 precursor protein*) *gene* [4]. Additionally, recent genetic studies have identified novel risk loci associated with AD, including genes involved in lipid metabolism, endosomal trafficking, and immune response, further underscoring the complexity of the disease [4].

These insights are crucial for developing new therapeutic strategies targeting not just Aβ and tau, but also inflammation and synaptic resilience. The non-invasive therapeutic approaches, FUS and TPS, are summarized in this review. Also discussed are the adipose-derived stem cells (ADSCs), gene therapy, and other alternative methods (diet, sleep pattern, physical exercise, nanoparticle delivery) that reduce inflammation and can be added to the conventional therapies.

## 2. Pathological Aspects of Alzheimer’s Disease

AD is defined by the insidious appearance of symptoms, such as progressive cognitive and functional decline, followed by neuropsychiatric symptoms based on the accumulation of Aβ plaques, NFTs, and neuron loss. In addition to the classically described changes, AD implies other pathological processes such as inflammation, vascular changes, mitochondrial dysfunction, and synaptic abnormalities [5]. Recent findings have identified neuroinflammation as a turning point contributor to AD progression, with chronic activation of microglia and astrocytes aggravating neuronal impairment. Elevated levels of pro-inflammatory cytokines, such as Interleukin-6 (IL-6) and Tumor Necrosis Factor α (TNF-α), have been associated with both the early and late stages of AD [6]. AD dementia is manifested by significant and progressive disabilities throughout the disease, with death occurring within 5–12 years of the onset of symptoms [7]. After baseline, younger people had a longer life expectancy than older people, but they had a greater reduction in life expectancy compared to the general population of the same age. In addition, men had shorter life expectancy than women [2,8]. The presence of two APOE ε4 alleles or a higher level of education are considered risk factors in AD regarding faster disease progression and an earlier death [2]. At the same time, in a study of the memory of a cohort with AD, the median duration of prodromal AD was three years. Still, they did not make any age-specific estimation. The most accurate prognosis in AD evolution is given by the both amyloid and neuronal injury markers, as proposed by the National Institute of Ageing-Alzheimer Association criteria [9]. The increased t-tau in CSF in patients with prodromal AD showed a faster conversion to AD dementia [10,11]. 

Macroscopic changes in AD are characterized by cortical atrophy with enlarged sulcal spaces and atrophy of the gyri, especially to the level of the precuneus and posterior cingulate gyrus [12]. Recent high-resolution imaging studies have shown that atrophy patterns in these regions strongly correlate with tau deposition in the medial temporal lobe, as detected through advanced tau-PET imaging [13]. Memory deficits in AD are linked with reductions in the volume of the hippocampus and amygdala, with crucial roles in memory, decision-making, or emotional response [14]. Neuronal loss has been observed in the cortical associative areas of the frontal, temporal, and parietal lobes or the subcortical nuclei (e.g., the noradrenergic locus coeruleus, the cholinergic basal nucleus, and the serotonergic dorsal raphe) [15]. Serrano et al. highlighted the loss of neuromelanin pigmentation in the locus coeruleus [16], a pontine nucleus involved in responses to stress and panic. New studies suggest that the degeneration of the locus coeruleus may precede other pathophysiological changes in AD, highlighting its potential role as an early biomarker for the disease [17]. Although these changes are not always typical, their presence, associated with clinical manifestations and lack of confirmation for other neurodegenerative diseases, supports AD diagnosis.

Microscopically, the brain of AD is characterized by the loss of neurons and synapses based on the presence of amyloid plaques and NTFs. These pathological changes are often associated with inflammatory phenomena and brain capillary alterations [18]. A recent study confirmed that the capillary alterations involve pericyte degeneration, which disrupts the BBB and intensifies neuroinflammation, linking vascular pathology with cognitive decline [19]. There is information underlying the vascular hypothesis of neurodegeneration in AD [20]. According to this theory, early triggers of homeostatic misbalance are the common vascular risk factors as age, hypertension, and cholesterol. The results include atherosclerotic disease and microvascular fibrosis followed by functional consequences, such as chronic hypoperfusion, which initiates neurovascular remodeling cascade. The aberrant clearance of Aβ across the BBB initiates the accumulation of Aβ in the brain and brain vessels, and the endothelial senescence and angiogenic response support increased Aβ. Defective adaptive angiogenic response perpetuates chronic hypoxia, jointly leading to increased Aβ burden and clinical symptoms of mild cognitive impairment. Subsequent accelerated synapse loss and neurodegeneration in combination with progressive vascular pathology results in advanced cognitive loss characteristic of progressive disease [20].

The cholinergic neurons from the cerebral cortex are lost due to the drastic decrease in their functional and morphological features [21,22]; similar atrophy of neurons is observed in the nucleus basalis of Meynert [23]. Cuello and Sofroniev concluded that AD is secondarily produced by retrograde atrophy of cholinergic neurons from the basal nucleus, which can be treated by applying Nerve Growth Factor (NGF) [24], with the maintenance of the “cholinergic tone” [25,26].

Aβ protein is the main molecule associated with amyloid plaques; it has a neurotoxic role produced by the protease cleavage of type I transmembrane APP [27]. APP cleavage occurs through two main pathways (amyloidogenic and non-amyloidogenic), as well as alternative pathways, which can generate APPα, APPβ, Aβ peptides, and intracellular APP, each leading to different outcomes [28]. Although the role of APP cleavage is not fully understood, evidence suggests that it influences neural development by affecting cell fate specification and neurogenesis in neural stem cells during brain development [29].

Amyloid plaques accumulate in the extracellular space, while the abnormally phosphorylated tau accumulates within neurons, forming abnormal filamentous tangles [30]. The tauopathy from AD consists of the development of insoluble aggregates as a consequence of hyperphosphorylated tau protein, which can spread in different regions of the brain [31]. Current literature emphasizing the dynamic nature of tau pathology shows that the process begins focally and expands due to the Aβ and neuronal connections, strongly influenced by synaptic activity [32,33,34,35,36]. One study assessed temporal tauopathy’s cortical origin and early progression using PET imaging [33]. From the initial location on the entorhinal cortex, the aggregates spread into the neocortex of the temporal lobe and then into the extra-temporal regions, and the expansion is correlated with neurodegeneration and cognitive decline [33].

Molecular pathway analysis of AD suggests the multitude of genetic components involved. In late onset, 58–79% of cases are with hereditary transmission, and in early onset, this transmission reaches over 90% [37]. Three genes are very well known for early onset: *APP*, *PSEN1*, and *PSEN2*, coding for amyloid-precursor protein, presenilin-1, and presenilin-2. The vast majority of cases are late onset. The ε4 allele of the *apolipoprotein E* (*APOE*) gene is linked to an increased risk of developing AD, making it the first gene identified as a significant risk factor for the condition [38]. Applying proof confirms that carriers of two copies of the APOEε4 allele face up to twelve times greater risk of manifesting late-onset AD compared to non-carriers, while carriers of one ε4 allele show a three-fold increase in risk. A 2023 genome-wide association studies (GWAS) meta-analysis highlighted the role of rare APOE variants, such as *APOE ε4* homozygous mutations, which may influence disease onset and progression differently than previously understood [39]. Furthermore, researchers have identified protective roles of the ε2 allele in mitigating AD risk [40]. More genes involved in the late onset are the *calcium homeostasis modulator protein 1* (*CALHM1*) [41] and the *translocase of outer mitochondrial membrane 40* (*TOMM40*) [42]. Other genes were discovered to promote the development of AD: *clusterin* (*CLU*), *complement receptor type 1* (*CR1*), *phosphatidylinositol binding clathrin assembly protein* (*PICALM*), and *alpha-2-macroglobulin* (*A2M*) [43,44,45].

The involvement of the triggering receptor expressed on myeloid cells 2 (TREM2), a gene associated with microglial function, has recently garnered attention. Variants of *TREM2* have been shown to impair the clearance of Aβ plaques and amplify neuroinflammation, linking it more strongly to AD pathogenesis [46].

The interest in the genes’ potential implications in the development of AD led to the polygenic risk scores that can nowadays predict the disease with high accuracy. GWASs and other sequencing projects identified more than 40 AD-associated loci [47]. By 2023, updated GWAS data had identified over 70 loci implicated in AD, revealing more genes involved in lipid metabolism, immune function, and tau processing. This advancement enables the use of polygenic risk scores in clinical trials to stratify patients based on genetic risk profiles [48].

However, in vitro experiments primarily focus on APP and tau processing-related genes.

Dourlen et al. considered as intervening factors in a hypothetical amyloid cascade the implication of *sortilin-related receptor 1* (*SORL1*), *Fermitin-family homolog 2* (*FERMT2*), *APOE*, *PICALM*, and cluster of *differentiation 2* (*CD2*)*-associated protein* (*CD2AP*) *genes* in APP metabolism; *APP*, *PSEN1*, *PSEN2*, and *Aph-1b protein* (*APH1B*) are linked to the APP production (amyloidogenic pathway) [47]. At the same time, the Aβ peptide clearance is under the control of *APOE*, *adenosine triphosphate* (*ATP*)*-binding cassette transporter* (*ABCAT*), *CLU*, and *PICALM genes* referred to the blood–brain clearance, while *TREM2*, *APOE*, *phospholipase C gamma 2* (*PLCy2*), *Abelson interactor* (*ABI3*), and *CD33 genes* intervene in microglial clearance [43].

A 2022 detailed study concerning the involvement of microglial cleavage in AD development showed that genes involved in lysosomal function and autophagy, such as *granulin* (*GRN*) and *transmembrane protein 106B* (*TMEM106B*), significantly influence the ability of microglia to clear Aβ deposits, thus raising the issue of potential identification of new therapeutic targets [49]. *CLU* and *PICALM genes* determine neuronal toxicity, but, more importantly, the tau metabolism is controlled by *Bridging Integrator 1* (*BIN1*), with the production of NTFs [46] and GRB-associated binding protein 2 (GAB2) [50].

The most important genes involved in AD development are presented in Table 1.

Other genetic factors are implied in the memory loss and neurodegeneration observed in AD (e.g., the disrupted dominant–negative protein kinase Mζ (PKMζ) observed in disturbed memory [35] or striatal-enriched phosphatase (STEP) in the synaptic dysfunction); others regulate endocytosis, cholesterol transport, protein ubiquitination, and innate immunity [51,52,53,54]. The genetic predictors of the pathology may be sex-related, with one locus on chromosome 7 having a sex-specific association with NFTs in males and giving them specific protection from tau pathology [54].

Pre-existing pathologies are an important key factor in the occurrence of AD. As was already mentioned, age is a key factor in AD development. It is demonstrated that accelerated aging exacerbates tauopathies [55]. The study pointed out the presence of tau phosphorylation in the amygdala and hippocampus.

Obesity is a risk factor for hypertension, diabetes, and cerebrovascular diseases. All these pathologies are known as indirect factors for cognitive decline. There is strong evidence that vascular risk increases AD probability [56]. A 2022 review emphasized the role of vascular endothelial dysfunction as an early marker of AD, showing that obesity-related inflammation accelerates amyloid and tau pathology [57]. In association with these pathologies or alone, obesity is correlated with a reduction in white matter, especially in the limbic system and in the area of connection between the temporal and frontal lobes [58].

Regardless of the age and mode of AD onset, it is certain that the mental state is affected by the degree of education and socio-economic status. The higher they are, the lower the risk of transition from normal Mini-Mental State Examination (MMSE) to mild MMSE [59]; it seems that cognitive health is maintained by education and life experiences. Recent studies support this theory, showing that individuals with higher cognitive reserve and multilingual abilities exhibit a delayed onset of clinical symptoms despite significant AD pathology [60].

## 3. Treatment

### 3.1. Current Treatment

Nowadays, treatment of patients with AD benefits from different types of medication used to stabilize mental performance, alleviate behavioral problems, prevent further brain damage, and basically, cope with everyday life. Cholinesterase inhibitors (medications that inhibit the degradation of acetylcholine) have been approved for mild and moderate dementia; they can help stabilize mental performance within a year. Additionally, they can carry out routine activities and continue performing daily tasks. However, the medication will not prevent or stop the progression of the disease (e.g., the loss of nerve cells, the increase in Aβ and activated microglia) [61,62].

Most of the medication in use today has been introduced before 2003: Tacrine was approved by the U.S. Food and Drug Administration (FDA) in 1993, but because its liver toxicity is no more prescripted [63]; Donepezil (Aricept^®^) in 1996; Rivastigmine (Exelon^®^) in 1998; Galantamine (Razadyne^®^) in 2001; Memantine (Namenda^®^) in 2003 (available in the U.S. since 2004). Most of these drugs function as cholinesterase inhibitors, which means that they block the acetylcholinesterase and are prescribed for mild to moderate symptoms; this ensures increased levels of acetylcholine in the brain that might improve the memory, speaking ability, thinking, or certain behavioral symptoms and, in some patients, could assist with daily tasks. The beneficial effects are only for several months to several years, and the disease progresses continuously. Memantine acts on the NMDA receptors, inhibiting their overactivation, counteracting the adverse reaction of increased glutamate levels in the brain. In this context, memantine protects neurons against glutamine-mediated excitatory toxicity [62].

The use of biomarkers in clinical research has facilitated the development of biomarker-based disease models. A common model emphasizes that different pathologic features of AD do not arise simultaneously but rather co-evolve in a staggered offset manner. Specifically, AD’s biomarker abnormalities begin with those of amyloid, followed by tau, and afterward by neurodegeneration. Clinical symptoms are the last in the sequence, many years after the onset of biomarker-evident amyloidosis, and symptoms are most closely linked to tau and neurodegeneration [1].

Recent advances have introduced therapies targeting amyloid plaques, which represent a significant shift in AD treatment. In June 2021, Aducanumab (Aduhelm™, ADU) became the first drug from the modern class of Aβ antibodies to receive approval from the FDA. Nowadays, the efficacity of Aducanumab is controversial because of its conflicting trial results [64].

Lecanemab, an anti-amyloid monoclonal antibody, received FDA approval in 2023 for patients with mild cognitive impairment (MCI) or early-stage AD. Clinical trials demonstrated that Lecanemab slowed cognitive and functional decline by 27% compared to placebo over 18 months [62]. In July 2024, the FDA approved Donanemab, another amyloid-targeting monoclonal antibody, for early symptomatic AD. Donanemab showed the potential to reduce amyloid plaques effectively and delay disease progression, particularly when initiated in the early stages of the disease [65]. While these advancements mark a milestone in the management of AD, they come with challenges. Both Lecanemab and Donanemab require intravenous administration and close monitoring for adverse effects (e.g., amyloid-related imaging abnormalities or ARIA), and are associated with high costs, which may limit accessibility for many patients. Despite these limitations, these drugs represent a new era in AD treatment, complementing existing symptomatic therapies.

Recent studies found that polysaccharides (PSs) potentially benefit in AD, in in vitro and in vivo studies. In 2021, the results of a 36-week phase 3 clinical trial of sodium oligomannate for mild to moderate Alzheimer’s dementia demonstrated significant efficacy in improving cognition with sustained improvement across all observation periods. Sodium oligomannate GV-971 was safe and well tolerated [66]. PSs may mitigate pathogenic damage and ameliorate cognitive symptoms by (1) counteracting the toxicity of aberrant tau and amyloid-beta proteins, (2) reducing oxidative stress and pro-inflammatory responses, and (3) restoring neuroplasticity [67].

Current molecular therapies of AD are synthesized in Table 2.

Continuing research and clinical trials are expected to refine these approaches and introduce additional options for addressing the underlying pathophysiology of AD.

### 3.2. Non-Drug Therapies

Despite the extensive research and clinical trials, several new possible therapies have failed to meet the efficacy endpoints or to show a drug/placebo difference, or they have shown unacceptable toxicity (e.g., small molecule therapy and immunotherapies) [5].

The rapidly ongoing technical and clinical progress opens new perspectives for AD therapies. Since brain diseases are one of the most critical problems of the rapidly aging population, the new therapies with undoubted results are needed. However, medication is not the only approach to treating AD. Since the 1950s, ultrasound therapies have been applied to the brain [68]. Nowadays, focused ultrasound (FUS) has been under discussion for therapeutic applications in neurodegenerative diseases. A new variant of FUS, transcranial pulse stimulation (TPS), works by sending ultrashort ultrasound pulses to small brain areas. TPS is a clinical sonication method based on a single ultrashort ultrasound pulse, while FUS techniques utilize acoustic wave propagation. Recent studies have provided evidence of TPS’s potential benefits for AD. A 2022 clinical trial demonstrated that TPS can safely improve cognitive function in patients with mild to moderate AD. The study indicated that TPS enhances neuroplasticity and regional brain connectivity, potentially slowing the progression of neurodegeneration [69]. Furthermore, a 2023 follow-up study confirmed the sustained cognitive benefits of TPS, reporting improvements in memory retention and executive function after repeated sessions [52].

Since stem cell therapies have been used in clinical practice for several years, they were eventually investigated as a possible cure for AD. Adipose tissue-derived stem cells (ADSCs) are now considered one of the best-suited cell types to play a key role in regenerative therapies [70]. They were involved in clinical studies for cell-based treatments because of their possible functions in replacing or repairing dead or damaged brain cells. A 2022 phase I trial found that direct transplantation of ADSCs into the hippocampus of AD patients was safe and showed preliminary signs of efficacy, including better memory performance and reduced Aβ plaque density [71].

More recent research also supports the promise of ADSCs for Alzheimer’s therapy. In 2023, a study using ADSC-derived exosomes reported reduced neuroinflammation and improved synaptic function in AD mouse models [72].

An attractive specific and effective treatment is represented by gene therapy. Researchers are interested in gene therapy as a potential treatment for AD. Replacing a gene that is missing or defective can be another method to save brain cells, keeping them healthy or generating new cells to reduce disease. As a result, a stable or inducible expression of the therapeutic gene can occur in targeted cells, protecting the brain cells from dying and preventing memory loss and cognitive decline. Recent advancements in gene therapy have further strengthened its potential as a treatment for AD. For instance, a 2023 study utilized CRISPR-Cas9 technology to successfully downregulate tau protein expression in a mouse model, significantly reducing the formation of NTFs and improving cognitive outcomes [53]. Similarly, viral vector-based gene delivery was used to enhance brain-derived neurotrophic factor (BDNF) production in the hippocampus, which promoted neuronal survival and improved memory in AD animal models [73].

**Figure 1:**
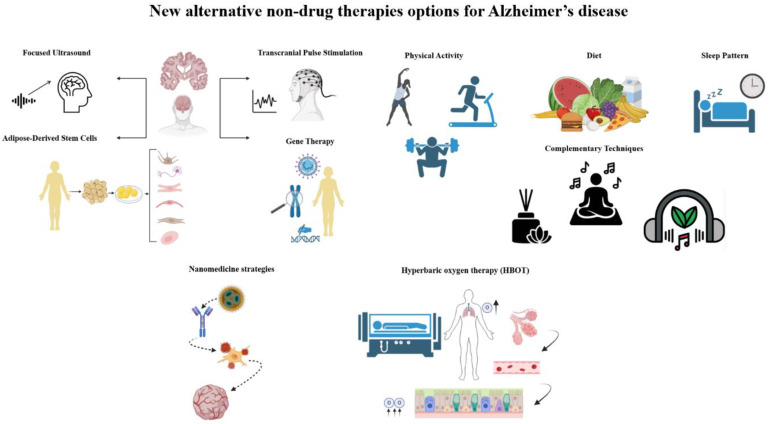
New alternative non-drug therapeutic options for AD.

Alternative therapies under investigation include nanomedicine strategies, oxygen therapy, physical activity, diet, sleep patterns, and complementary techniques such as occupational therapy, psychotherapy, aromatherapy, and music therapy [74] (Figure 1).

#### 3.2.1. Focused Ultrasound

Ultrasounds are acoustic waves above 20 kHz, a higher frequency than the upper audible limit of human hearing, that can be precisely targeted, influencing specific and selective regions. The bony structure of the skull, which protects the brain, strongly attenuates the transmission of waves [75], which leads to the need to use a large amount of energy. However, exposure to high-energy sound fields heats rapidly, quickly reaching unacceptable temperatures [76].

Applying different sonication protocols—considering the fundamental elements of sonication as frequency, pulse repetition frequency, intensity, duty cycle, and duration—can have profound effects on the brain [77]. Researchers stimulated the somatosensory cortex [78] or the motor cortex [79], with possible future applications in movement disorders [80], the visual cortex [81], or the hippocampus to obtain synaptic plasticity [82]. On the other hand, ultrasounds help to release molecules to knock out regular brain activity [83] or for neuromodulator purposes [84]. Thus, stimulation with high spatial resolution allows new non-invasive techniques to treat human brain disorders.

The administration of drug therapies in the pathology of the central nervous system (CNS) is generally affected by the structure of the brain and the molecular structure of the drug. More than twenty years ago, FUS was thought to be a possible method to improve medicine distribution to the brain by opening the BBB [85].

The BBB is a selective semipermeable structure between the systemic circulation and nervous parenchyma of the CNS, playing both a protective and a regulatory role in the molecule transfer from the bloodstream to the brain. The capillaries are formed by the endothelium, pericytes, and smooth muscles cells. The endothelial cells interact with the astrocytes surrounding the capillaries, neurons, and microglial cells. Between all these cells, there are reciprocal interactions and, together with the extracellular matrix, form a neurovascular unit (NVU), an efficient regulatory system of the brain. NVU links the neuronal activities to blood flow, and regulates the BBB permeability, the glymphatic system, and neuro-vascularization. NVU dysfunction leads to different brain pathologies, the epidemiological studies showing a high degree of comorbidity among vascular disease and dementias, including Alzheimer’s disease [20,86].

The BBB is crucial in maintaining brain homeostasis, allowing diffusion and active transport of solutes and nutrients, ions, and macromolecules between the capillary blood and brain tissue [87]. Due to its structure, the BBB prevents the passage of pharmacological molecules to obtain therapeutic concentrations in the nervous tissue. Therefore, the BBB serves as a target for therapeutic development in nervous pathology [88]. Advances in medical techniques have given the possibility to influence certain nervous structures by avoiding lesions of the surrounding tissues. Acting on the BBB, the ultrasounds disrupt the structure in a non-invasive manner, generating a temporary therapeutic window to enable the large molecules to cross [88,89]. The interference of the sonic waves with endothelial cells increases the permeability of the BBB, including the breaking of tight junctions between endothelial cells, increased transcytosis, and a reduction in drug efflux mechanisms [90]. Combining FUS with microbubbles (MB), the interference of FUS with endothelial cells implicates the development of a cellular cavitation. In general, the microbubbles (1–4 μm) have a lipid or albumin shell, filled with air, sulfur hexafluoride, or perfluorocarbons [90]. FUS-MB induced a mechanical BBB opening post-cavitation associated with increased permeability. The consequences are molecular, cellular, and structural changes [91,92]. MB cavitation described the main physical mechanism that generates effects from hours to weeks. Numerous studies have reported the optimal FUS-MB experimental parameters, underlying the MB parameters (e.g., type, dosage, size, injection methods) or FUS parameters (e.g., frequency, pressure, burst parameters, duration) or targeted brain region and pathological conditions. The developing tools used to monitor and assess the treatment improve FUS targeting accuracy and demonstrate its short- and long-term safety. Its first application was reported by Lehman and Herrick [89]. The infusion of contrast agent MB (FUS-MB) improves the BBB’s temporary opening [93]. In the AD animal model, relevant to the effect of the FUS, is the repetitive application of FUS-MB weekly or biweekly for 4–10 weeks, in safe doses, with no side effects but with regression of Aβ plaque more effectively compared to a single FUS-MB application [94]. In a recent 2023 clinical study, MRI-guided FUS (MRIgFUS) successfully opened the BBB in multiple regions of the brain in human AD patients [95]. Another study, using sub-micron bubbles (nanobubbles), with long circulation time, demonstrated the BBB opening similar to the opening obtained by the microbubbles in use [96].

This therapeutic approach implied as a premise the efficacy of Aβ antibodies in treating AD. In 2006, Kinoshita et al. demonstrated, for the first time, the applicability of FUS in administering antibodies to the brain [97,98]. They find the circulating antibodies in the brain after BBB permeabilization by FUS.

However, the administration of the antibodies in the presence of amyloid is still controversial due to the changes in the properties and functions of the BBB. In the beginning, the studies focused on the safety of the BBB opening in AD mice. The studies revealed that the BBB changes produced by FUS were comparable in transgenic mice AD (*PS1/APP*) and wild-type control [98]. In a similar study with *PDAPP* transgenic mice, it was demonstrated that the BBB opening was comparable in aged mice, 12 vs. 26 months, despite increased skull fragility and altered vascularity described in older transgenic mice [99]. A 2024 study further confirmed that BBB opening via FUS remains effective and safe across diverse AD animal models, regardless of age. These results open possibilities for the use of FUS in elderly human populations, which was previously a concern due to increased vascular fragility [100].

Kinoshita et al. also determined (by trypan blue stained plaques) that anti-Aβ antibodies’ presence in the brain followed their administration into the bloodstream due to the BBB opening by FUS [85,97]. New findings from 2023 demonstrated that BBB disruption using FUS not only facilitates the delivery of therapeutic anti-Aβ antibodies and other large molecules but also induces microglial activation, enhancing the brain’s innate immune response and significantly reducing the amyloid plaque burden without adverse effects [95,101].

Sun et al. suggest that in mice, the delivery of a monoclonal antibody anti-pyroglutamate-3 amyloid-β (pGlu3 Aβ) (a modified, pathogenic form of amyloid-β found in amyloid plaques and vascular deposits) using the BBB permeabilization by FUS-MB, led to great plaque removal (increasing monocyte infiltration in the plaque), saving the synapses, and improving the cognitive function with no apparent damage [102]. A 2023 study further supported this finding, demonstrating that FUS-mediated delivery of pGlu3 Aβ antibodies led to a 45% reduction in amyloid plaque density in mouse models. The study also confirmed improved synaptic plasticity and significant cognitive enhancements in treated animals [103].

A single or repetitive application of FUS-MB treatment induced BBB opening, allowing the entry of endogenous immunoglobulin, Ig G, and IgM, the activation of glial cells, especially the microglial cells, reducing the Aβ plaque and p-tau with positive consequences in the memory and cognitive deficits [104,105,106]. Another mechanism initiated by FUS-MB repetitive application is the autophagy-mediated pathway and glymphatic–lymphatic pathway for the Aβ and p-tau clearance [107]. Recent research has highlighted that repetitive FUS-MB applications not only facilitate amyloid clearance but also improve glymphatic flow in animal models of AD. Improved glymphatic activity was associated with reduced neuroinflammation and better cognitive outcomes, offering a dual benefit for AD management [100]. Stimulating the glymphatic system and enhancing CSF flow through low-intensity FU (LIFUS), another variant of FUS stimulation, can also boost brain function in AD disease models. A 2023 study demonstrated that repetitive LIFUS applications in mice enhanced the clearance of amyloid-beta (Aβ) plaques and tau aggregates, promoting synaptic recovery and memory improvements [108].

The MRI-guided FUS (MRIgFUS), the most commonly used technique, allowed the delivery of larger molecules (glycogen synthase kinase-3 (GSK-3) [109], anti-tau antibodies [110], nanoparticles [111]) with therapeutic effects. Also, there are studies demonstrating that a single FUS-MB application facilitates the entrance to the brain of the Aβ antibodies as Aβ antibody (BAM-10) [112] or anti-Aβ protein antibody (BC-10) [113], and intravenous immunoglobulin (IVIg) [114]. All molecules react with Aβ plaque, and the targeted region has less Aβ plaque. A 2023 clinical study demonstrated that MRIgFUS enables the safe delivery of nanoparticles specifically designed to target tau aggregates in AD. This targeted approach showed a significant reduction in tau pathology and improved cognitive function in early-stage AD patients [100]. These studies demonstrated the feasibility of targeting Aβ plaques and p-tau present in the brain with bloodstream-administered antibodies that cross the BBB after transcranial FUS.

Another variant of FUS stimulation using low-intensity pulsed ultrasound (LIPUS) targeting the whole brain, one hemisphere, or the hippocampus also contributes to the reduction of Aβ plaque [115,116]. The repetitive LIPUS application activated the glial cells, directly [103] or by upregulation of endothelial nitric oxide synthase (eNOS) [117], reducing Aβ plaque. On the other hand, the repetitive LIPUS application could stimulate neurogenesis by increasing the expression of neurotrophic factors or could increase cholinergic activity, thus improving memory and cognitive deficits [116,117]. In 2023, a study demonstrated this novel mechanism of action for LIPUS stimulation; it improved the expression of BDNF and synaptic proteins in the hippocampus of AD mice. This neurogenesis-promoting effect was accompanied by a marked reduction in Aβ plaques and tau tangles [118].

All these small animal studies reveal the efficacy of BBB opening via FUS (or FUS-MB) in a non-disruptively confined area. To have better confirmation of the safety of using FUS, Pouliopoulos et al. investigated the cellular and behavioral risk profile following BBB opening in non-human primates [100]. Using a clinical prototype of a neuronavigation-guided system, they identified a brief immune response in the targeted area, increased microglia density on day 2 (resolved by day 18), and enhanced immature neurons around BBB-open areas. The BBB opening did not deteriorate cognitive performance.

The results are similar in a study for recurrent glioblastoma treatment, using a device combining neuronavigation and a manually operated frameless FUS system [119]. The result was a BBB opening in a dose-dependent manner, with no immunological response. The study was completed with the application of a higher safe dose of FUS in a rat glioma model, leading to the recruitment of lymphocytes into the tumor microenvironment (TME) and transforming the immunosuppressive TME into an immunostimulatory one [119].

In human studies, the parameters of FUS included a central frequency of 220 kHz, sonication power of 4.5–4.6 W, 3.6–7.5 sonications for 300 ms (each spot with 2 ms on and 28 ms off), and Definity MB infusion (4 μL/kg) [120,121,122,123]. The result is an opening of BBB without side effects such as death, hemorrhages, swelling, or neurological deficits.

In mild to moderate AD patients, the repetitive MRIgFUS-MB application in 2–3 treatment sessions produces persistent glymphatic efflux after BBB opening [120], with a transient decrease in frontoparietal neuronal function, restored after 24 h [121], and no clinical changes detected within 1 to 3 months after treatment [122,123]. This was recently confirmed by a 2023 clinical trial that validates the safety and efficacy of repetitive MRIgFUS-MB treatments in AD patients. The study reported persistent BBB permeability improvements, enhanced glymphatic clearance, and measurable reductions in amyloid burden over a 6-month period without adverse neurological outcomes [124].

The first described study of FUS application in AD patients was a study by Nicodemus et al. in 2019. Under control of MRI and Doppler ultrasound, they applied one-hour FUS stimulation using a 2 MHz transducer at a power of 520 mW/cm^2^, targeting the mesial temporal lobe. The effect was an improvement in cognitive function for 63% of patients and an improvement in motor functioning after 8 weeks of therapy for 9.1% of patients. The patients also presented increased perfusion of the targeted region [125].

Until now, none of the available antibody medications targeting Aβ plaques showed full potential in treating AD. However, given the therapeutic potential of ultrasound in AD treatment, if an effective antibody therapy becomes available, FUS could have a synergistic effect.

In a prospective phase II clinical study exploring MRI parameters after FUS-BBB opening, Mehta et al. observed a selective BBB opening accompanied by the enhancement along the venous drainage immediately and 24 h after sonications. The authors’ interpretation was that the blood–meningeal barrier permeabilization is a side effect of BBB opening, with implication in immune cells circulation [126].

**Figure 2:**
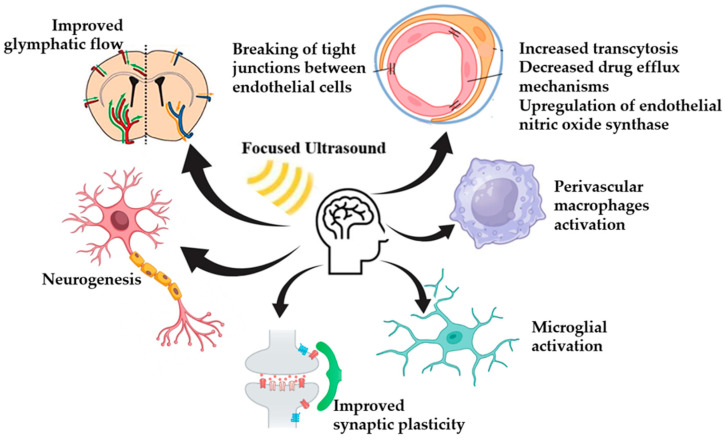
FUS application bioeffects.

To conclude, FUS application opens the BBB and facilitates the therapeutic molecule therapy. There is also evidence for several secondary effects of BBB opening by FUS, such are activation of inflammatory pathways, neurogenesis stimulation, and cell-specific activation. The bioeffects of FUS application are summarized in Figure 2.

For a precise FUS application in specific brain region, MRI guidance can be used. Its use is limited when the FUS target is represented by deep structures such as the globus pallidus, which is not visible in conventional 3Tesla MRI [127].

Circumspections of FUS application, in the first instant, are given by the frequency of sonications, the total number of safe administrations, the long-term effects, and the risk of repeated BBB disruption [128]. Another limitation is given by the targeted region due to the skull and scalp heterogeneities which alter the ultra-sound propagation. These heterogeneities combine the density of cortical bone, bone marrow, and the skull thickening. In these conditions, the US waves are reflected, especially at the high frequencies. Using lower frequencies may reduce this effect, but increases the risk of tissue damage [119,129,130]. Because of its thickening, the human skull induces a bone attenuation of USs 20-fold-higher than the soft tissue [131]. Related to the amount of energy transferred to the brain, US application may produce local heating and/or local cavitations (especially in the use of US contrast agents or in the existence of local gas bodies), sometimes followed by cell damage and local hemorrhages [132,133,134].

The improvement of FUS parameters can be ensured by new soft- and hardware development.

#### 3.2.2. Transcranial Pulse Stimulation

An important limitation of brain stimulation was the duration of the neuromodulator effects, which was reduced to a few minutes. As a result, researchers had to focus more on the long-lasting changes in the brain during the stimulation itself, with no harmful changes, which could be useful in the therapy of different pathologies [135,136]. Thus, TPS is a new non-invasive technique, based on single ultrashort ultrasound pulses (3 µs, repeated every 200–300 ms). TPS is characterized by a spatially distinct stimulation, being able to stimulate the deep brain structures (e.g., thalamus) [137]. TPS generates ultrashort pressure pulses consisting of multiple frequencies with higher amplitude [135,136]. The application of very short pulse, instead of periodic waves or long sonication trains, avoids tissue heating and standing waves [135,136,138,139].

Beisteiner et al., in 2019, presented the results of the first multicenter clinical trial using TPS [135,136]. The study was simultaneously performed in two countries, Austria and Germany, and its observations of long-term effects were provided. Feasibility, safety, and efficacy data are available, consisting of simulation evidence, comparative laboratory measurements between rats and human skulls and brains, and in vivo modulations of somatosensory evoked potentials (SEP) in healthy subjects. Clinical data from 35 patients with AD, including neuropsychological scores and functional magnetic resonance imaging (fMRI) images, were acquired. During the study, the patients received ongoing and optimized standard clinical treatment [136]. The Consortium to Establish a Registry for AD (CERAD) corrected total score (CTS), as an attested measure of cognitive state in AD, was visibly improved after treatment and remained stable for three months. CERAD principal component analysis (PCA) explores functional brain connectivity, allowing for monitoring of the separate cognitive components for memory (MEMORY), verbal processing (VERBAL), and visuospatial processing (FIGURAL). The treatment was followed by interesting results, with a dissociation between the parameters. While the MEMORY and VERBAL performances were improved, the FIGURAL performance was depressing. This last result was presented by center 1 (Austria), which, compared to center 2 (Germany), did not stimulate the parieto-occipital cortex, an area that contributes to visuospatial processing [136]. The results pointed out the idea of obtaining specific therapeutic effects related to stimulated areas/networks. At the same time, the CERAD results highlight the significant improvement in the subjective evaluation of memory performance (SEG). Also, in the analysis of depression scores, it was shown that the neuropsychological improvement was not determined by the changes in the depressive mood [136].

In a 2023 extension of Beisteiner’s trial, researchers followed 50 AD patients over 12 months, observing continued improvements in cognitive performance, as measured by CERAD-CTS and logistic regression scores. The study highlighted a longer-term effect of TPS on memory and verbal fluency, with additional fMRI data showing improved connectivity in the parieto-occipital cortex [140]. The CERAD logistic regression score (LR), a measure of AD dementia, was significantly improved and remained stable for more than three months as a result of the treatment [140].

In 2019, Verhagen et al. demonstrated that long-lasting changes could be achieved through repetitive ultrasound stimulation, targeting deep areas within a primate’s brain while avoiding structural damage [141]. They found a uniform activation of the stimulating area, even in rest and strongly interconnected areas; reduced activity was observed in areas with little or no interconnection. Thus, TPS intervenes in the circuits between close areas and could constitute the mechanism through which specific regional changes can be induced to determine behavioral changes [142]. Therefore, ultrasound techniques unequivocally sustain reversible changes in the neural circuits of the primate brain [143]. This approach can develop TPS protocols in research or clinical intervention.

A follow-up study concluded that TPS moderates cortical atrophy in AD by modulating the cortical thickness of the stimulated brain [144]. This has been found in specific regions known to be very important for AD patients: the default mode network. This network degenerates very early during AD, having an important role in cognitive and memory performance. A 2023 study confirmed the potential of TPS in modulating functional connectivity in the brain’s default mode network, showing that TPS reverses cortical thinning in key regions of the default mode network, contributing to enhanced cognitive function (improvements in memory retention, visuospatial ability, and neural network coherence) in AD patients [145].

Two other follow-up studies (2021, 2024) demonstrated that TPS reduces amyloid burden and tau aggregates in AD animal models. The ultrasound stimulation pulsed at 40 Hz decreases, especially insoluble Aβ (Aβ40 and Aβ42 levels, completely) in the cerebral cortex and hippocampus, with evident functional improvement [146,147]. In the 2024 study, the intervention was also associated with increased hippocampal synaptic density and improved spatial memory performance [146]. These findings provide additional evidence for the therapeutic role of TPS in mitigating AD pathology.

Another important topic is how ultrasounds produce specific neuronal changes and can generate neuroplastic effects. Current knowledge in this field is limited but related to ultrasound-based techniques, with several principles being proposed. Most likely, mechanical effects are produced on the cell membranes that affect the mechanosensitive ion channels and generate membrane pores. As a result, the concentrations of the neurotransmitter and the humoral factor may change. Extracellular serotonin and dopamine levels are increased, and the level of Gamma-Aminobutyric Acid (GABA) is decreased, in parallel with the increase of BDNF, vascular endothelial growth factor (VEGF), and glial cell line-derived neurotrophic factor (GDNF). All these molecules can produce changes at the cellular network level [99].

A recent study corroborates these findings, confirming that TPS also modulates neurotransmitter release in AD models. Researchers observed increased levels of dopamine, serotonin, and brain BDNF in treated animals, correlated with improved synaptic plasticity and cognitive resilience [148].

The use of ultrashort ultrasound pulses in neural stem cell cultures has led to increased stem cell proliferation and neuronal differentiation [149].

Other studies have shown in an AD mouse model that microglial activation is associated with plaque reduction, the clearance of Aβ into microglial lysosomes, and improvements in spatial and recognition memory, or they highlight the role of NOS in improving cognitive dysfunctions associated with dementia through whole-brain stimulation [150,151]. These findings are not in contradiction with criticisms of the Aβ model.

**Figure 3:**
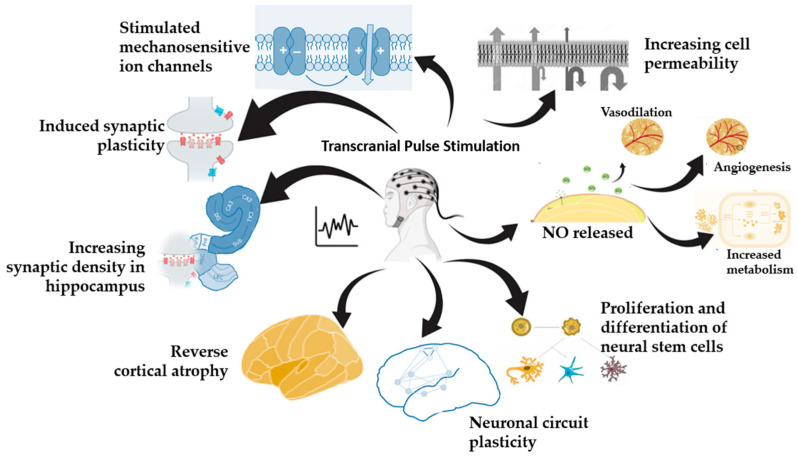
Biological effects of TPS.

The biological effects of TPS are induced by the mechanotransduction [152]. The mechanical stimulus is converted into biochemical responses which affect the cell functions. First, the stimulus promotes growth factor expression, particularly VEGF [153,154], which improves cerebral blood flow and stimulates angiogenesis and nerve regeneration. Additionally, it releases nitric oxide (NO) [155], which leads to vasodilation, increased metabolic activity, angiogenesis, and anti-inflammatory effect. The effect on the stem cells includes the proliferation and differentiation of neural stem cells [156]. Other effects are increasing of cell permeability [157], modification of the level of the neurotransmitters and humoral factors [158], and increasing the expression of brain-derived neurotrophic factor (BDNF) [159]. The biological effects of TPS are summarized in Figure 3.

In conclusion, the data confirm that therapeutic TPS is beneficial for treating AD, demonstrating both functional effects (shown in animal and human studies) and morphological effects (such as reducing cortical atrophy). The TPS technique can be spatially distinct and highly focal, and is not restricted to superficial layers of the brain [127]. Minor side effects were reported, such as headache, uncomfortable feeling, pain, and pressure, but 80–90% of patients reported no sensation of pain or pressure during TPS treatment, and more than 60% reported no side effects after TPS [127,160,161]. However, the published results discussed a crucial consideration, which is the substantial variability in the efficacy of TPS demonstrated across clinical TPS trials [162]. The small sample size of the included studies and the considerable heterogeneity of protocols and study populations within them represent a challenge in the comprehensive interpretation of the results.

#### 3.2.3. Therapy with Adipose-Derived Stem Cells

As is already known, ADSCs are now widely used in the therapy of various diseases, including other neurodegenerative disorders (e.g., Huntington’s and Parkinson’s disease, stroke) [163,164]; their characteristics ensure a high therapeutic potential in regenerative medicine [165,166,167]. Located in the stromal vascular fraction and resembling in aspect mesenchymal stem cells, they possess a strong proliferation capacity (easily favored and maintained by different growth factors and cytokines) and in vitro multilineage differentiation (various types of conjunctive cells, muscular fibers, neurons, etc.) [168]; they are easy to collect from the ubiquitarian adipose tissue (an abundant and accessible source of stem cells in the body at all ages) [168,169], and therefore they have the advantage of being cheap and biocompatible, and, very importantly, they can be cultivated under standard conditions [165,166,167,168]. Also, their extensive secretome (with antiapoptotic, hematopoietic, angiogenic, and immunomodulatory substances) and immunobiological properties are favorably implicated in tissue repair and regeneration [165,166,167]. However, until the end of 2021, neither standardized protocol on human patients (regarding the growth and isolation of ADSCs or quality assurance) nor clinical trials with this therapy have been described in the literature. The different methods used in laboratories were reflected in donor differences (impacting the ADSC profiles that are extremely heterogeneous), reagent properties (sources, quality), cell culture, and isolation techniques [167,168,169,170,171]. All this information makes it extremely difficult to draw a pertinent prognostic conclusion.

A 2024 review emphasized this need for standardized protocols for ADSC isolation, characterization, and quality control in clinical applications for AD. This study also called for further investigations into the optimal dosing, administration routes, and long-term safety of ADSCs in human trials [172].

Two recent studies (2023) highlighted the potential of ADSC-derived exosomes in reducing amyloid-beta plaques and tau hyperphosphorylation in transgenic AD mouse models. These exosomes were shown to cross the BBB and deliver neuroprotective molecules, including VEGF and BDNF, resulting in significant improvements in cognitive and memory functions [69,173].

Additional models of great rigor that reiterate the AD are still needed for an accurate perspective concerning this concept. The animal models (mouse/rat) are considered experimental systems of great value. They aim to replicate the in vivo AD human condition due to their genetic and genomic similarities with humans (identical deoxyribonucleic acid (DNA) in more than 80% [174]). The Novel Object Recognition Test (NORT) represents a common, rapid, and relatively accurate way of testing the ability of rats and mice to learn and memorize [175]. It was validated as an evaluating tool for quantifying the modifications of the learning and memory processes induced by drug administration or by genetic and neurological interventions. This test is based on three sessions: animals’ habituation and training and their testing of the different phases of learning and memory; NORT was adapted successfully for rodents, which have an innate preference for novelty [175].

A 2023 study applied NORT in ADSC-treated transgenic AD mice and demonstrated significant improvements in short-term memory and recognition tasks compared to untreated controls. The study also confirmed enhanced synaptic density and reduced neuronal loss in the hippocampus, further validating the effectiveness of ADSCs in reversing cognitive deficits [32].

The efficiency of exogenous stem cell transplantation therapy is still controversial, mainly due to the low grafting efficiency of exogenous stem cells in the brain [176,177]. On the other hand, stem cell therapy is able to produce the specific neurons and glial cells, potentially replacing damaged or lost neural cells in different neurological pathologies. But the memories and cognition are encoded in engrams, the neurons’ circuitry, and the new neurons are not recovering the engrams [178,179,180].

#### 3.2.4. Gene Therapy

Gene therapy is an option that can lead to a stable and specific expression in the targeted cells. There are two ways genes are delivered: across the BBB, increasing its permeability using FUS-MB in targeted places, or modified gene carriers, which can circumvent the BBB and deliver genes directly into the brain cells. An important advantage of gene therapy is the persistence of gene expression after one therapy administration.

Gene therapy can be applied by a vector, in general, a virus that infects the host cells. The direct results of gene therapy consist of increased enzymatic activities and compensation for the reduced levels of bioactive substances. Different viral vectors have been developed in neurodegenerative diseases, such as AD (e.g., the recombinant adeno-associated viral vector rAAV and the Human Immunodeficiency Virus (HIV)-derived lentiviral vector). In the neuron, a non-dividing cell, gene expression in the infected cell can persist for a long time, even up to 6 years [181].

Many experiments demonstrated the implication of nervous system growth factors in preventing neuronal death in animal models of AD [25,182,183]. The relevant growth factors for AD are the NGF and BDNF.

In 2018, Rafii et al. published data that proved the viability of placebo surgery-controlled stereotactic gene delivery studies in AD patients. Adeno-associated viral vector (serotype 2)—NGF (AAV2-NGF) delivery was viable and well tolerated, but yielded no clinical outcomes [184].

The first clinical trial was initiated in 2001 by Tuszynski et al. when 10 patients with AD underwent gene therapy to overexpress the *NGF gene* [185,186]. The results indicated that the degenerated neurons could respond to growth factors by axonal growth, cellular hypertrophy, and the activation of functional markers, phenomena persisting over ten years. Other studies used the implantation of microcapsulated patient-derived fibroblasts, genetically modified to overproduce NGF [183,184]. The various results included an increased arborization in basal forebrain cholinergic neurons (BFCN), intensification of glucose metabolism at the cortical level, and the normalization of cholinergic markers in CSF, but without a substantial improvement in cognitive decline [185,186,187,188].

A 2024 trial evaluated NGF gene delivery via an updated AAV2 vector in AD patients. The results showed significant improvements in glucose metabolism and markers of neuroinflammation but only modest effects on cognitive function. This suggests that NGF-targeted therapy may be more effective as part of a combination treatment strategy [189].

New research has expanded the potential of AAV-based delivery systems. A 2023 study explored the use of AAV9 to deliver BDNF in animal models of AD. The study demonstrated significant cognitive improvements, increased synaptic density, and reduced amyloid burden in treated animals [190]. This approach highlights the emerging role of neurotrophic factors in AD treatment through gene therapy.

Another study used a *peroxisome proliferator-activated receptor gamma coactivator 1-alpha gene* (*PGC1-alpha gene*) released by a modified virus in the brain cells of mice. This study is based on a previous study of the authors, where it was demonstrated that *PGC1-alpha* regulates the transcription of β-APP cleaving enzyme (BACE1), the main enzyme involved in Aβ generation, with a decreased expression in AD patients. The results consist of a reduction in the development of AD, with no depletion of brain cells in the hippocampus; improved memory; and minimal amyloid plaques after four months of injection. The group of mice who were not treated had more plaques in their brains [191].

Another important gene therapy implicates the *APOE4 gene*, which is responsible for the production of APOE proteins [184]. APOE genes provide information for the synthesis of APOE, which, combined with fatty molecules, forms lipoproteins. Lipoproteins are responsible for the transport of lipids and the metabolism of lipids. There are three known variants of the *APOE genes—APOE2*, *APOE3*, and *APOE4*—which are present in 7%, 79%, and 14%, respectively, in the general population. The *APOE2 gene* is associated with type III hyperlipoproteinemia but has an anti-AD effect; the risk of developing AD is reduced by half. *APOE3* is considered the healthy gene. *APOE4* increases the risk of developing AD by promoting higher levels of amyloid proteins in the brain [192].

The reverse of *APOE4* into *APOE3* is the target for researchers from the Massachusetts Institute of Technology [193]. The process used skin-derived pluripotent stem cells to convert them into three different types of brain cells: neurons, microglia, and astrocytes. These cells were then used to replace unwanted genes, which were cut down on bacteria that act as molecular scissors.

In 2023, another study using CRISPR-Cas9 technology successfully converted the *APOE4 gene* into the *APOE3* variant in neuronal cultures derived from AD patients. This gene-editing technique reduced amyloid plaque accumulation and restored normal lipid metabolism pathways, marking a promising therapeutic approach for addressing the genetic risks associated with AD [194].

Recently has been applied the antisense therapy to block tau production. The results of a randomized clinical trial, published in 2023, evaluated the effect of tau synthesis reduction on tau biomarkers in patients with mild AD. BIIB080, an antisense oligonucleotide that targets *MAPT* pre-messenger RNA and reduces tau synthesis associated with cognitive decline in dose-dependent manner [195].

There are many approaches in trying to treat AD, such as CRISPR (as was seen before, the perfect tool for gene disruption), but also theranostics (using both diagnosis and therapy tools as part of the treatment), peptidomimetics (using an analog of natural proteins exhibiting equivalent or higher biological effects in interaction with biological targets), etc.

Much must be done before it becomes a routine practice, but the results of all studies demonstrated what a powerful arm this therapy is in the fight against AD.

### 3.3. New Alternative Non-Drug Therapies

#### 3.3.1. Nanomedicine Strategies

Various nano-biomedicine therapies have been studied to enable targeted delivery to specific areas affected by AD while preserving brain integrity and successfully crossing the BBB [196].

For example, a study conducted on the brain of AD animal models using polymeric nanomicelles with the property of freeing levels of 3D6 antibody fragments (3D6-Fab) showed suppression of Aβ aggregation [197]; also, innovative immunotherapy approaches employing single-chain anti-Aβ antibodies (scFv) notably decreased Aβ accumulation in an acute amyloidosis model [198].

Metallic nanoparticles show promising drug delivery properties that are useful in managing AD. Gold nanoparticles (Au-NPs), in particular, demonstrate great permeability properties through the BBB. When conjugated with glutathione, Au-NPs inhibit Aβ aggregation, potentially preventing AD progression. Silica nanoparticles (SiNPs) are also used for BBB targeting, accumulating in the amyloid cells and demonstrating a potential anti-AD effect [199].

#### 3.3.2. Oxygen Therapy

It is well known that hypoxia represents a key pathogenetic factor in AD, promoting amyloid-ß metabolism, tau phosphorylation, autophagy, neuroinflammation, oxidative stress, endoplasmic reticulum stress, and mitochondrial and synaptic alteration [200]. It seems that anti-hypoxia therapies could slow down or diminish AD evolution [200]. Published data have revealed that most pathogenic features of AD were ameliorated [40,200,201,202]. Growing evidence in recent years has highlighted the beneficial role of oxygen therapy concerning the risk factors and clinical symptoms of AD [40,200,201,202]. Thus, oxygen therapy could represent a useful therapeutic option for this disease.

**Figure 4:**
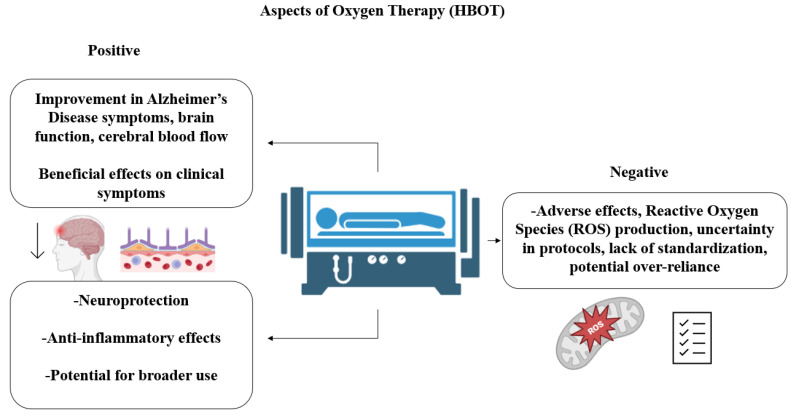
Positive and negative effects of HBOT.

Hyperbaric oxygen therapy (HBOT) is a medical intervention in which patients breathe 100% oxygen through a mask or head tent within a hyperbaric chamber, which is pressurized to between one and three atmospheres. HBOT has been suggested for different disorders, such as carbon monoxide poisoning, decompression sickness, post-concussion syndrome, chronic wound healing, radiation injuries, stroke and neurodegenerative illnesses, aging, and dementia or surgery preoperative preparation. The effects of the reactive oxygen species (ROS) on AD are closely correlated with the number of treatments, duration, and pressure, which may lead to diverse oxidative stress situations. The processes triggered by HBOT are linked to elevated levels of dissolved oxygen and enhanced oxygen pressure. In HBOT, the fraction of inspired oxygen is augmented, which increases the proportion of dissolved oxygen in the blood and in tissues by approximately five to twenty times. The excess oxygen can spread into areas that erythrocytes cannot access; also, the partial pressure of oxygen increases, which produces hyperoxia. While short-term HBOT causes the production of ROS, long-term HBOT improves mitochondrial function, decreases the levels of ROS, and increases the antioxidant protective mechanisms. ROS are essential for the cellular substrates involved in memory and also play a role in impaired neuroplasticity during senescence and AD [203,204]. ROS control N-methyl-d-aspartate (NMDA) receptors, calcium and potassium channels, and the Ca^2 +^/calmodulin kinase II mechanism. HBOT reduces vascular dysfunction, amyloid burden, brain inflammation, BBB integrity, hippocampal TNF-α synthesis and neuronal apoptosis, dendritic spine loss, astrocyte activation, pro-inflammatory acute phase proteins, interleukins, and cytokines. The anti-inflammatory effect of HBOT could be mediated by hyperoxia interfering with Nuclear Factor κB (NF-κB) and IκBα [203,204,205,206]. HBOT increases growth factors, pro-angiogenesis cytokines, parasympathetic nervous system activity, vagal stimulation, arteriolar luminal diameter and cerebral blood flow, degradation, and clearance of Aß protein. As a result, HBOT improves cognitive function, memory and recognition, neurogenesis, and synaptogenesis and modulates cortical alpha rhythm. HBOT also could produce a structural change in blood vessels that reduces brain hypoxia. However, HBOT is associated with several adverse effects, including anxiety from confinement, middle ear, and pulmonary barotrauma, air embolism, temporary loss of consciousness, hypotension, myopia, bradycardia, and a disconnect between heart rate and cardiovascular system responses (Figure 4).

There is still no consensus on a standardized HBOT protocol for different neurological diseases, including the optimal air pressure, duration, and frequency of treatment sessions, that would yield maximal neurological benefits with minimal side effects. Imaging techniques, such as PET and MRI, may offer further insights into the effects of HBOT on degenerative brain diseases [205,206,207].

#### 3.3.3. Physical Activity

Recent data have revealed that physical activity not only diminishes the frequency of AD, but moderate physical exercises have beneficial roles in functional and cognitive abilities, improving the life quality of AD patients [196,208].

Current evidence from the scientific literature highlights that sports reduce the negative influence of brain amyloid on cognition, diminished cerebral atrophy, and the incidence of dementia by improving cardiovascular fitness in AD patients [168]. Mainly, aerobic exercise had a beneficial effect on cognitive functions and depression of AD subjects, being associated with better cerebral blood flow and occipital gray matter density [208,209].

Despite the unknown mechanism, persistent physical exercise seems to prevent declines in patients with mild and severe forms of AD [208]. Moreover, exercising increases the physical health of AD patients, diminishing the behavioral and psychological manifestations of dementia and improving their daily living activities [209].

Supporting its protective role against AD, several studies revealed that physical exercise produced cerebral structure modifications like upregulation of cerebral neurogenesis and an enlarged hippocampus [208].

The beneficial role of sports in AD patients could be explained by several mechanisms: the augmentation of the Aβ-40 and Aβ-42 clearance and the suppression of the BACE-1 activity, the lowering of tau hyperphosphorylation by enhancing the activity of γ-secretase [210,211,212], the increased utilization of glucose and insulin tolerance [213], and the diminishing of pro-inflammatory cytokines like IL-6 or TNF-α [214].

Amplifying proof continues to reveal the positive roles of persistent and strong exercise in AD, improving the advancement of indispensable activities of daily tasks and cognitive functions due to enhanced neuroprotective factor levels [215,216,217].

#### 3.3.4. Diet

The Mediterranean diet and nutrition supplements improve cognitive function and prevent AD due to their neuroprotective and antioxidant properties [218]. Studies have shown that a diet rich in docosahexaenoic acid (DHA) and eicosapentaenoic acid (EPA), folic acid, and vitamins B6, B12, E, C, etc.) improved cognitive function and diminished the accumulation of Aβ and cerebral atrophy [74,208,218,219]. The beneficial role of vitamin B in AD is supported by the inhibition of homocysteine, a metabolite responsible for increased neurodegeneration, via enhanced oxidative activity [219].

Consumption of food rich in omega-3 fatty acids [220] and essential amino acids (Val, Leu, His, Lys, Trp, Ile, Phe) improved cognition and psychosocial function [221]. Furthermore, nutritional supplements derived from plants with anti-inflammatory, antioxidant, and neuroprotective functions, such as scallop-derived purified plasmalogen [222], curcumin, epigallocatechin-3-gallate, resveratrol or probiotics, improved cognitive activity in mild AD [223,224,225].

A study on AD mice using niacin supplements demonstrated that niacin could increase cognitive activity in AD, representing a useful preventive and therapeutic option for AD patients [226].

Current research has revealed the beneficial role of combined metabolic precursors (CMAs), composed of Nicotinamide Adenine Dinucleotide (NAD^+^) and glutathione precursors, in cognitive activity in AD patients [227].

#### 3.3.5. Sleep Pattern

Evidence highlights that sleep activity has an important impact on AD pathogenesis, influencing the behavioral, cognitive, and memory dysfunctions of this illness [74]. Sleep alterations produce a high amyloid accumulation due to an inflammatory mechanism, increasing the abovementioned processes [228]. In this regard, several studies have shown that the reduction of melatonin with neuroprotective properties is involved in the initial phases of AD [229]. Melatonin suppresses the accumulation of APP and Aβ by regulating the secretase activity [229,230,231,232]. Moreover, it improves the neurotoxicity produced by Aβ via stimulating Aβ elimination through glymphatic–lymphatic drainage and degradation ways [229,230,231,232]. Thus, melatonin benefits cognitive activity and sleep patterns, representing a potential therapeutic strategy for AD [232]. Moreover, other therapeutic options to ameliorate sleep quality are being investigated, for example, melatonin agonists such as ramelteon [233], agomelatine, tasimelteon [234], and bright light therapy [235].

#### 3.3.6. Complementary Techniques

Alternative complementary therapies like aromatherapy and music seemed to improve the usual clinical manifestations of AD patients, such as behavioral symptoms of anxiety and depression [74,236].

In addition, occupational therapy and psychotherapy play an essential role in the therapeutic approach to AD [237]. Occupational therapy aims to improve the autonomy of these patients, allowing the development of usual activities through cognitive and behavioral exercises [237,238]. The main role of psychotherapy in patients with AD emerges from the fact that it can give them confidence and strength to cope with emotional disturbances, depression, or anxiety [238].

## 4. Conclusions

The high failure incidence of AD therapies highlights the intricate and multifaceted pathological causes of the disease, as well as the gaps in our understanding of how the numerous biological pathways involved in its development interact. This includes the progression of neurodegeneration and the potential lack of effectiveness of existing therapeutic agents. We have reached a turning point in the approach to treating AD. Beyond pharmacological treatment, FUS/TPS, ADSCs, and gene therapy have significant potential. Despite intensive research in that field, the fact that there is still no medication to stop or reverse AD’s progress should make us wonder if we should not pay more attention to other therapeutic concepts, sometimes less conventional.

During the last decades, most drugs tested in studies of AD therapies have identified specific molecular targets; besides preventing Aβ accumulation as the main mechanism, new targets include oxidative stress, inflammation, or glial activation. Even RNA-based therapeutics could become a direction in AD treatment. Methods that aimed at reducing behavior disturbances were investigated in some clinical studies. Generally, AD therapies address one pathogeneic pathway, but due to the complex and interconnected pathways, treatments of AD face significant limitations.

In the future, targeting multiple pathways (with medical and alternative methods) with synergic action may be required for a successful treatment.

## Figures and Tables

**Table 1:** The most important genes involved in AD development.

**AD Onset**	
Early onset	APP, PSEN1, PSEN2
Late onset	APOEε4
**AD Linear Evolution**	
APP metabolism	APP, PSEN1, PSEN2, ADAM10, SORL1, FERMT2, APOE, PICALM, CD2AP, APH1B, A2M
Aβ clearance through the BBB	CLU, ABCAT, PICALM, APOE
Aβ clearance through microglia	APOE, TREM2, PLCy2, ABI3, CD33, GRN, TMEM106B
Tau metabolism	BIN1, GAB2
Neuronal toxicity	CLU, PICALM

**Table 2:** Current molecular therapies.

AD Current Treatment		
Cholinesterase inhibitors	mild to moderate dementia	Donezepil Rivastigmine Galantamine
NMDA Glutamate receptor antagonist	moderate to severe dementia	Memantine
Aβ antibodies	early-stage of dementia	Aducanumab Lecanemab Donanemab
Polysaccharides	mild to moderate dementia	Sodium oligomannate GV-971

## Abbreviations

3D6-Fab	3D6 antibody fragments
AAV2-NGF	Adeno-associated viral vector (serotype 2)—NGF
ABCAT	ATP-binding cassette transporter
ABI3	Abelson interactor3
AD	Alzheimer’s disease
ADI	Alzheimer’s disease international
ADSCs	Adipose-derived stem cells
APH1B	Aph-1b protein
APOE	Apolipoprotein E
APP	Amyloid beta precursor protein
ARIA	Amyloid-related imaging abnormalities
ATP	Adenosine triphosphate
Au-NPs	Gold nanoparticles
Aβ	Β-amyloid
BACE1	Β-APP cleaving enzyme
BFCN	Basal forebrain cholinergic neurons
BIN1	Bridging integrator 1
Cas9	CRISPR-associated protein 9
CD	Cluster of differentiation
CD2AP	CD2-associated protein
CERAD	Consortium to Establish a Registry for Alzheimer Disease
CLU	Clusterin
CMAs	Combined metabolic precursors
CNS	Central nervous system
CR1	Complement receptor type 1
CRISPR	Clustered regularly interspaced palindromic repeats
CSF	Cerebrospinal fluid
CTS	Corrected total score
DHA	Docosahexaenoic acid
DNA	Deoxyribonucleic acid
eNOS	Endothelial nitric oxide synthase
EPA	Eicosapentaenoic acid
FDA	Food and Drug Administration
FERMT2	Fermitin family homolog 2
FIGURAL	Visuospatial processing
fMRI	Functional MRI
FUS	Focused ultrasound
FUS-MB	Focused ultrasound combined with the infusion of contrast agent microbubbles
GABA	Gamma-aminobutyric acid
GDNF	Glial cell line-derived neurotrophic factor
GRN	Granulin
GSK-3	Glycogen synthase kinase-3
GWAS	Genome-wide association studies
HBOT	Hyperbaric oxygen therapy
HIV	Human immunodeficiency virus
Ig	Immunoglobulin
IL-6	Interleukin-6
IVIg	Intravenous immunoglobulin
IκBα	Inhibitor of nuclear factor kappa B
LIFUS	Low-intensity focused ultrasound
LR	Logistic regression score
MEMORY	Cognitive components for memory
MMSE	Mini-mental state examination
MRIgFUS	Magnetic resonance imaging -guided focused ultrasound
NAD	Nicotinamide adenine dinucleotide
NF-κB	Nuclear Factor κb
NfL	Neurofilament light chain
NFTs	Intracellular neurofibrillary tangles
NGF	Nerve growth factor
NMDA	N-methyl-d-aspartate
NORT	Novel object recognition test
NVU	Neurovascular unit
p-tau	Phosphorylated tau
PCA	Principal component analysis
PET	Positron emission tomography
PGC1-alpha	Peroxisome proliferator-activated receptor gamma coactivator 1-alpha
pGlu3 Aβ	Monoclonal antibody anti-pyroglutamate-3 amyloid-β
PICALM	Phosphatidylinositol binding clathrin assembly protein
PKMζ	Protein kinase Mζ
PLCy2	Phospholipase C gamma 2
PS1	Presenilin 1
PSEN1	Presenilin gene 1
PSEN2	Presenilin gene 2
rAAV	Recombinant adeno-associated viral vector
ROS	Reactive oxygen species
scFv	Single-chain anti-Aβ antibodies
SEG	Subjective evaluation of memory performance
SEP	Somatosensory evoked potentials
SiNPs	Silica nanoparticles
SORL1	Sortilin related receptor 1
STEP	Striatal-enriched phosphatase
t-tau	Total tau
TME	Tumor microenvironment
TMEM106B	Transmembrane protein 106B
TNF-α	Tumor necrosis factor α
TREM2	Triggering receptor expressed on myeloid cells 2
VEGF	Vascular endothelial growth factor
VERBAL	Verbal processing
